# Health status and socio-economic factors associated with health facility utilization in rural and urban areas in Zambia

**DOI:** 10.1186/1472-6963-12-389

**Published:** 2012-11-13

**Authors:** Cosmas Zyaambo, Seter Siziya, Knut Fylkesnes

**Affiliations:** 1 Department of Community Medicine, School of Medicine, University of Zambia, Lusaka, Zambia; 2 School of Medicine, Copperbelt University, Ndola, Zambia; 3 Centre for International Health, University of Bergen, Bergen, Norway

**Keywords:** Zambia, Health care seeking, Wealth index, Educational attainment, Self-rated health

## Abstract

**Abstracts:**

**Background**

With regards to equity, the objective for health care systems is “equal access for equal needs”. We examined associations of predisposing, enabling and need factors with health facility utilization in areas with high HIV prevalence and few people being aware of their HIV status.

**Methods**

The data is from a population-based survey among adults aged 15years or older conducted in 2003. The current study is based on a subset of this data of adults 15–49 years with a valid HIV test result. A modified Health behaviour model guided our analytical approach. We report unadjusted and adjusted odds ratios and their 95% confidence intervals from logistic regression analyses.

**Results**

Totals of 1042 males and 1547 females in urban areas, and 822 males and 1055 females in rural areas were included in the study. Overall, 53.1% of urban and 56.8% of rural respondents utilized health facilities past 12 months. In urban areas, significantly more females than males utilized health facilities (OR=1.4 (95% CI [1.1, 1.6]). Higher educational attainment (10+ years of schooling) was associated with utilization of health facilities in both urban (OR=1.7, 95% CI [1.3, 2.1]) and rural (OR=1.4, 95% CI [1.0, 2.0]) areas compared to respondents who attained up to 7 years of schooling. Respondents who self-rated their health status as very poor/ poor/fair were twice more likely to utilize health facilities compared to those who rated their health as good/excellent. Respondents who reported illnesses were about three times more likely to utilize health facilities compared to those who did not report the illnesses. In urban areas, respondents who had mental distress were 1.7 times more likely to utilize health facilities compare to those who had no mental distress. Compared to respondents who were HIV negative, respondents who were HIV positive were 1.3 times more likely to utilize health facilities.

**Conclusion**

The health care needs were the factors most strongly associated with health care seeking. After accounting for need differentials, health care seeking differed modestly by urban and rural residence, was somewhat skewed towards women, and increased substantially with socioeconomic position.

## Background

There is an increased awareness of the inequality in access to healthcare services [1] and this has renewed the government’s commitment to improve the health status of the poor [2]. The main objective of most international organizations is to improve the health outcomes in the poor [3-5] which has resulted in an increased tendency to define their goals in terms of poverty reduction [4,6] and in a broader interpretation of what poverty really means [7-9]. It reflects a growing consensus that inequality in the use of health services and health outcomes are unjust and unfair [10]. This does not mean that the poor are more deserving than the rich are, instead the inequalities evidently correspond to the widely differing constraints and opportunities rather than a tendency to make different choices [11-14].

Health services research has a long tradition in evaluating inequalities in access to health care and guiding policies to promote equitable access, and in this regard, health surveys have played an important role [15]. A behaviour model of health services use has frequently been used to guide multivariate analyses of health care visits [16]. The model has evolved over time and has considered visits, particularly, those initiated by the individual, to be a consequence of predisposing, enabling, and need variables. In this model, ‘predisposing’ refers to demographic factors such as social status, education, and beliefs; ‘enabling’ is the ability of an individual to secure services through income, health insurance, and community factors such as availability of health care services; and ‘need’ refers to the perceived illness or health status. The model is mainly used to test the impact of factors, other than ‘need for care’, on the utilization of health care services [16].

Like many sub-Saharan countries with substantial inequalities in income and access to resources such as health care, Zambia has the objective of ‘equity of access to cost-effective quality health care as close to the family as possible’ [17]. There exists different type of healthcare services namely professional services which comprises of the public health sector and private health sector, and the traditional health sector which includes different types of traditional practitioners. The public health sector is financed and supervised by the government under the ministry of health; this sector is organized nationally on the basis of a pyramidal referral structure, consisting of the central, provincial and district hospitals and health centers. There are three health referral systems, the primary level which receives the patients from the health centers and refers the patients to the secondary level. The secondary level refers to the third (tertiary). The Public sector offers the advantage that care is available both in urban and rural areas on the other hand the private health sector are mostly private clinics (for profit) and church affiliated (missions) hospitals. The ‘for profit’ private clinics provide clinical services care on a fee-for-service and are not controlled or financed by the government. A few are financed on the basis of long term contracts with the formal sector employers [17-19]. The church affiliated health institutions are an important component of Zambia’s health system as well; they are the second largest providers of health services in the country after the government hospitals. There are a number of reasons why users might use the private sector even when public facilities are available, suggested reasons could be ease of geographic access, short waiting period, long and more flexible opening hours, greater availability of staff and drugs or greater confidentiality in dealing with diseases [17-19].

There are 973 rural and 237 urban health centres in Zambia. The urban health centres serve approximately 30,000 to 50,000 people, and the rural health centres serve approximately 10,000 people in a radius of 29 km. Zambia has 72 districts and each has a district hospital that serves as the referral hospital for 200,000 to 800,000 people. There are also central hospitals that serve as specialised centres for 19 second level hospitals. Some of the health centres and hospitals are run by private institutions [18]. The health facilities within the reach of most people are the health centres. Patients must be referred to the next level in order to avoid paying a penalty. In Zambia, the primary reasons for hospital admission are HIV/AIDS co-morbidities, including tuberculosis, malaria, diarrhoea, and respiratory infections such as pneumonia [17].

For Zambia to develop a national policy with the objective of ‘equity of access to cost-effective quality health care as close to the family as possible’, there is a need to understand the factors that drive health care utilization; however, few studies have evaluated this issue [19,20]. To that end, we used data from a population-based HIV survey of selected urban and rural areas to investigate factors associated with utilization of health facilities.

## Methods

### Population-based survey

This cross-sectional HIV survey was conducted in 2003 in Chelston and in the rural part of Kapiri Mposhi district using a stratified random-cluster sampling method. Chelston is a suburb of Lusaka, which is the capital city of Zambia, and has a fairly static core population and relatively high population density, while the rural part of Kapiri Mposhi in the Central province has a low population density.

The Zambia census of the population mapping system was used to establish the sampling frame which consisted of all 26 standard enumeration areas (SEAs) in Kapiri Mposhi (5225 households) and all 24 SEAs in Chelston (2786 households). The SEA defined the primary sampling unit (cluster). The sampling design to select the standard enumeration areas was probability proportion to measure of size using the number of households in each area as derived from the 1990 census figures as a measure of size. In Chelston, the sampling size was 10 SEAs (44% of the households) and in Kapiri Mposh there were also 10 SEAs (40% of the households). In these selected 20 SEAs, all the household members of these households aged 15 and above were listed and invited to participate in the study [21,22]. In this study, we used a subset of this data comprising respondents who had valid HIV results and aged 15–49 years.

### Data collection

We collected information on socio-demographic characteristics, health status indicators, HIV status and related behavioural risk factors, and health care use. We scheduled an interview with the head of the household for enlisting eligible members, and then, we interviewed the eligible household members in order to collect information. Trained research assistants collected information in teams of 2 (1 man and 1 woman). Every respondent was informed about issues related to HIV testing (i.e. testing is based on blood and linked to counselling services). Voluntary counselling and testing services were offered and arranged for those who wished to know their HIV status, in accordance with established national guidelines of Zambia.

The Omni-SAL device (Saliva Diagnostic System, Inc., Singapore) was used to collect saliva. The specimens were transported once a week to a national reference laboratory (Lusaka University Teaching Hospital) and screened for HIV using Wellcome HIV-1&2 GACELISA (Wellcome Diagnostics, Dartford, Kent, UK). A total of 450 samples were randomly selected and retested using the BIONOR HIV-1&2 (BIONOR AS, Skien, Norway) magnetic particle-based-assays modified for saliva. Since there was a high degree of consistency between the 2 assays (99.8%), no further retesting was done.

### Measurements and statistical analysis

Analyses were performed using the Statistical Package for Social Sciences version 12 (SPSS for Windows; SPSS, Chicago, Illinois, USA) [23]. The intercooler Stata version 9.0 (College Station, Texas, USA) [24] was used to account for the cluster effect in this analysis. Logistic regression models were used to examine factors associated with health care utilization. Unadjusted odds ratios (OR), adjusted odds ratio (AOR) and their 95% confidence intervals (CI) are reported.

A participant was deemed to have utilized a health facility if he/she reported one or more visit to any health facility in the last 12 months. The behaviour model of health services use also includes educational status as a predisposing factor related to health care seeking behaviour, but since education is among the key indicators of socio-economic status, and thus relates closely to resources and opportunities, education was considered to have both a predisposing and enabling influence on health seeking behaviour. Other predisposing factors were age, sex, and marital status. Perceived health needs were measured using the dimensions of health status. Self-rated health was based on a single question, ‘How would you say your health is at the moment’? It was evaluated using a 5-point scale (excellent, good, fair, poor, and very poor). Excellent/good were combined and coded as 0, and very poor/poor/fair were coded as 1 [25-27]. This measure has been found to be a very sensitive marker of changes in the various dimensions of health status, to be among the strongest predictors of health care use, and to operate as a strong independent predictor of survival [28-30]. We evaluated the mental distress by using a self-reporting questionnaire (SRQ-10), which included 10 questions that had to be answered as ‘yes’, ‘no’, and ‘don’t know’. Very few participants responded ‘don’t know’and were considered missing. An additive index was constructed on the basis of the SRQ-10, and respondents with ‘yes’ on 3 or more of the questions were classified as mentally distressed. Self-reported illness was based on a three-part question: ‘in the last 12 months have you suffered from: (a) Malaria, (b) Tuberculosis, and/or (c) Sexual transmitted infections (STI). Participants who indicated that they suffered from any of these three illnesses were considered to have self-reported illnesses.

The enabling factors, other than educational status, were residence (urban vs. rural) and a wealth index based on the assets (electricity, refrigerator, radio, bicycle, donkey, and plough). The wealth index is considered a proxy indicator of the socio-economic status of a household [31-33]. The response was coded 0 if they did not have the asset and 1 if they had. The indicator variables were assigned weights. Considering electricity, refrigerator and radio, the rationale for weighting was that respondents cannot have a refrigerator if they do not have electricity. With this type of reasoning, respondents who had refrigerators were wealthier than those with electricity only. Radio was weighed less than electricity because it’s possible to have a radio without having electricity. A donkey is a means of transport and an agricultural asset, therefore, a respondent who had it was wealthier than the one who owned a plough. A respondent who had a radio was considered wealthier than one with the bicycle; the reasoning was that, a bicycle was a common mode of transport and most people owned bicycles but owning a radio was a luxury that required extra resources to spend on it. With this reasoning, the radio was weighed more than a bicycle. The summative approach of making an index was used. Using SPSS for computing, the assigned indicator weights were multiplied by the asset variable, and then the resultant score was obtained. The wealth index was divided into four quartiles and recoded into different variables to represent 1st quartile represents the poorest, 2^nd^ quartile-very poor, 3^rd^ quartile-poor, and 4th quartile-least poor as shown in (Table 1).

**Table 1:** Weights of the assets and the wealth index of the households

**Variables**	**Total=4466 n (% )**	**Weights**
**Assets**		
Refrigerator	2806 (52)	6
Electricity	3192 (59.2)	5
Radio	4103 (76.1)	4
Bicycle	1701 (31.5)	3
Donkey	24 (0.4)	2
Plough	576 (10.7)	1
**Household wealth/asset index**		
1st quartile (poorest)	1410 (26.2)	
2nd quartile	1323 (24.6)	
3rd quartile	2078 (38.6)	
4th quartile (least poor)	576 (10.7)	

### Ethics

We obtained ethical approval for the survey protocol from the University of Zambia Research Ethics Committee. Participants were counselled and informed that saliva-based testing was strictly for research purposes, and therefore anonymous. Those who showed interest in being counselled and tested were invited to contact a counsellor, and arrangements for collection of their blood samples were made.

## Results

### Participation

A total of 6,791 residents participated in the survey comprising 4,086 (1,861 male and 2,225 female) urban residents and 2,705 (1,301 male and 1,404 female) rural residents. Non-participation was because of absence (19.7%), interview refusals (3.4%), or refusal to provide a saliva sample for HIV testing (6.6%).

Out of a total of 4,466 respondents who had valid HIV results and aged 15–49 years, 1,877 (42.0%) lived in rural areas. Table 2 shows distributions of socio-demographic factors and health care utilization between urban and rural residences. More females (56.2% rural and 59.8% urban) than males participated in the study. Most of the respondents in urban areas were single (53.2%), while most of the respondents in rural areas were married (67.2%). Altogether, 85.2% of urban and 78.6% of rural respondents self-rated their health status as good/excellent. Overall, 53.1% of urban and 56.8% of rural respondents utilized health facilities in the previous 12 months to the survey.

**Table 2:** Socio-demographic characteristics and utilization of health facilities in urban and rural areas of Zambia (2003)

**Characteristic**	**Urban**	**Rural**
	**Total=2589 n (%)**	**Total=1877 n (%)**
**Predisposing factors**		
**Sex**		
Male	1042 (40.2)	822 (43.8)
Female	1547 (59.8)	1055 (56.2)
**Age of the respondents**		
15-19	745 (28.8)	369 (19.7)
20-24	718 (27.7)	396 (21.1)
25-29	446 (17.2)	334 (17.8)
30-39	456 (17.5)	491 (26.2)
40-49	226 (8.7)	287 (15.1)
**Marital status**		
Single	1500 (53.2)	470 (25)
Married	908 (35.2)	1256 (67.2)
Devoiced, separated, widow	171 (6.6)	142 (7.8)
**Enabling factors**		
**Education attainment**		
0-7	449 (17.4)	1273 (68.4)
8-9	472 (18.3)	301 (16.2)
10+	1660 (64.3)	287 (15.4)
**Household ownership**		
Electricity	2328 (90.1)	264 (14.1)
Radio	2289 (88.5)	1080 (57.7)
Refrigerator	2087 (80.7)	178 (9.5)
Bicycle	447 (17.3)	1009 (53.9)
Plough	89 (3.4)	403 (21.5)
Donkey	9 (0.3)	13 (0.7)
**Wealth index**		
Q1 (poorest)	191 (7.4)	990 (52.9)
Q2	438 (17)	699 (37.4)
Q3	1556 (60.2)	111 (5.9)
Q4 (least poor)	399 (15.4)	70 (3.7)
**Perceived health needs**		
**Self-rated health**		
Good/excellent	2196 (85.2)	1463 (78.6)
Very poor/poor/fair	382 (14.8)	398 (21.4)
**Self-reported illnesses**		
No	1339 (51.9)	606 (32.4)
Yes	1243 (48.1)	1265 (67.6)
**Mental distress**		
No	2239 (87.5)	1623 (87.1)
Yes	377 (12.5)	240 (12.9)
**HIV status**		
Positive	467 (18)	256 (13.6)
Negative	2122 (82)	1621 (86.4)
**Utilized health facility**		
No	1215 (46.9)	810 (43.2)
Yes	1374 (53.1)	1067 (56.8)

### Determinates of health facility utilization

#### Perceived health needs

Overall, self-rated health, mental distress, and self-reported illnesses were significantly associated with health facility utilization in both urban and rural areas (Table 3). Respondents who rated their health status as very poor/ poor/fair were twice more likely to utilize heath facilities in both urban and rural (urban, OR=2.0, 95% CI [1.5, 2.6]), rural OR=2.0, 95% CI [1.5,2.7]) compared to respondents who self-rated their health status as good/excellent. In urban areas, respondents who were mentally distressed were 1.7(95% CI [1.4, 2.4]) times more likely to utilize health facilities compared to respondents who were not mentally distressed. Respondents who reported having malaria, tuberculosis and/or sexually transmitted infections were thrice (OR=3.0, 95% CI [2.2, 3.9] in urban, and OR=3.6, 95% CI [2.9-4.4] in rural areas) more likely to utilise heath care services compared to those who did not report any of these illnesses. In rural areas, respondents who were HIV positive were 1.3 (95% CI [1.0, 1.9]) times more likely to utilize health facilities compared to those who were HIV negative.

**Table 3:** Logistic regression models showing the determinants of health care utilization in urban and rural areas in Zambia (2003)

**Characteristics**	**n (**^**1**^**%)**	**Urban**		**n (**^**1**^**%)**	**Rural**	
		**Unadjusted OR (95%CI)**	**Adjusted OR (95%CI)**		**Unadjusted OR (95%CI)**	**Adjusted OR (95%CI)**
**Age group**						
15-19	323 (23.7)	1	1	197 (18.5)	1	1
20-24	363 (26.6)	1.3 (1.0,1.6)	1.3 (1.0,1.6)	212 (19.9)	1.0 (0.7,1.3)	1.0 (0.6,1.3)
25-29	264 (19.4)	1.9 (1.4,2.4)	**1.7 (1.2,2.3)**	185 (17.4)	1.0 (0.8,1.4)	1.0 (0.6,1.4)
30-39	289 (21.2)	2.2 (1.7,2.8)	**2.0 (1.5,3.0)**	281 (26.4)	1.0 (0.8,1.5)	1.0 (0.6,1.4)
40-49	125 (9.2)	1.6 (1.3,2.1)	1.3 (0.8,1.9)	191 (17.9)	1.7 (1.2,2.4)	1.4 (0.9,2.2)
**Sex**						
Male	508 (37.2)	1	1	461 (43.2)	1	1
Female	856 (62.8)	1.3 (1.1,1.5)	**1.4 (1.1,1.6)**	605 (56.8)	1.0 (0.8,1.2)	1.2 (0.8,1.3)
**Marital status**						
Single	720 (52.8)	1	1	261 (24.5)	1	1
Married	535 (39.2)	1.5 (1.3,1.8)	1.0 (0.8,1.4)	706 (66.3)	1.0 (0.8,1.2)	1.0 (0.8,1.6)
Divorced/separated/widowed	105 (7.7)	1.7 (1.2,2.3)	1.0 (0.6,1.4)	98 (9.2)	1.7 (1.1,2.6)	**1.7 (1.0,2.8)**
**Educational status**						
0-7	211 (15.5)	1	1	658 (61.7)	1	1
8-9	234 (17.2)	1.1 (0.8,1.4)	1.2 (0.9,1.6)	203 (19.0)	1.9 (1.4,2.5)	**1.7 (1.2,2.3)**
10+	916 (67.2)	1.4 (1.1,1.7)	**1.7 (1.3,2.1)**	199 (18.9)	2.1 (1.6,2.8)	**1.4 (1.0,2.0)**
**Wealth index**						
Q1 (poorest)	89 (6.5)	1	1	518 (48.6)	1	1
Q2	221 (16.2)	1.1 (0.8,1.6)	1.0 (0.7, 1.6)	413 (38.8)	1.3 (1.0,1.5)	1.2 (0.9,1.5)
Q3	836 (61.3)	1.3 (0.9,1.8)	1.3 (0.9,1.9)	75 (7.0)	1.8 (1.2,2.8)	1.5 (0.9,2.5)
Q4 (least poor)	218 (16.0)	1.4 (0.9,1.9)	**1.5 (1.0, 2.2)**	59 (5.5)	4.8 (2.5,9.0)	**3.4 (1.6, 7.1)**
**HIV status**						
Negative	1066 (78.2)	1	1	884 (82.9)	1	1
Positive	298 (21.8)	1.7 (1.4,2.1)	1.1 (0.8,1.4)	182 (17.1)	2.0 (1.5,2.7)	**1.3 (1.0,1.9)**
**Self-rated Health**						
Good/excellent	1086 (79.1)	1	1	769 (72.1)	1	1
Very poor/Poor/fair	273 (20.9)	2.5 (1.9,3.1)	**2.0 (1.5,2.6)**	290 (27.9)	2.4 (1.9,3.1)	**2.0 (1.5,2.7)**
**Self-reported illness**						
No	525 (38.6)	1	1	200 (18.8)	1	1
Yes	836 (61.4)	3.1 (2.7,3.7)	**3.0 (2.2,3.9)**	865 (81.1)	4.3 (3.5,5.3)	**3.6 (2.9,4.4)**
**Mental distress**						
No	1137 (83.4)	1	1	900 (84.4)	1	1
Yes	209 (15.3)	1.8 (1.4,2.3)	**1.7 (1.4,2.4)**	163 (15.3)	1.7 (1.2,2.2)	1.2 (0.9,1.7)

All confidence intervals adjusted for cluster effect.

Associations in bold are statistically significant.

^1^% the percentage of respondents who went to any health facility.

#### Enabling factors

After adjusting for perceived health needs and predisposing variables, educational status and wealth index were significantly associated with health care utilization. In urban areas respondents who attained 10+ years of schooling were 1.7 (95% CI [1.3, 2.1]) times likely to utilise the healthcare services and in the rural areas respondents who attained 10+ years in school were 1.4 (95% CI [1.0, 2.0]) times more likely to utilise the healthcare services. Further, in the rural areas respondents who attained 8–9 years of schooling were 1.7 (95% CI [1.2, 2.3]) times more likely to utilize health facilities compared to those who had up to 7 years of schooling. Meanwhile, in rural areas, respondents who were in the highest wealth quartile were three times more likely to utilise health services than those in the lowest quartile (OR=3.4; 95% CI [1.6, 7.1]).

#### Predisposing factors

Overall, sex and age were significantly associated with health facility utilization in urban areas. Among urban residents, females were more likely to utilize health facilities than males (OR=1.4, 95% CI [1.1, 1.6]). In urban areas, compared to respondents who were in the 15–19 years age group, respondents in the age groups 25–29 years and 30–39 years were 1.7 (95% CI [1.2, 2.3]) and 2.0 (95% CI [1.5, 3.0]) times, respectively, more likely to utilize health facilities.

## Discussion

Data were obtained from a population-based survey conducted in areas with a high HIV prevalence and a low awareness of personal HIV status [34]. An important objective for health care systems is ‘equal access for equal needs’, and the main focus of our study was on the associations of place, age, sex, and socioeconomic position with utilization of health facilities after taking into account the individual need differentials being here measured as dimensions of health status. The public health services dominated the health care delivery in the areas studied. Visits differed modestly by urban and rural residence, but were somewhat skewed towards women. However, health care visits increased substantially with socioeconomic position (both with level of education and household wealth index) after adjusting for health care need. The most prominent associations were related to the need indicators.

Self-rated health has been found to be among the most sensitive indicators of the way individuals perceive various threats related to health or life stresses, and a large number of studies in high-income countries have consistently shown self-rated health to be the strongest independent determinant of health care use [27-30]. The results obtained from our data were consistent with these previous studies. Respondents who judged their health status as poor/fair were more likely to utilize health facilities than those who judged their health as good/excellent. It is important to note that HIV status was highly associated with self-rated health and that the effect of HIV status on seeking health care diminished sharply when adjusted for health status [27]. The most likely explanation is that the effect of HIV infection, in a setting where few know their HIV status, is mediated through self-rated health. This has previously been demonstrated in Zambian surveys in the same areas. A population-based survey revealed HIV infection to have a strong independent negative effect on self-rated health in persons older than 24 years. It was suggested that ‘this measure of people’s subjective health may be used as a valuable “diagnostic” tool in HIV-related care and support programmes and should be evaluated for use in such services’ [27]. Similar results were found in a randomised trial on voluntary HIV counselling and testing, which showed that self-rated health was the strongest determinant of readiness for testing [35]. A possible interpretation of this result was that people living in areas with a very high HIV prevalence perceive declining health status as a possible sign of HIV infection and that this in turn affects their health seeking behaviour. It has also been found that there is a strong effect of mental distress on seeking care especially in the urban areas. The plausible explanation could be that as the HIV disease progresses more people are affected with mental illnesses. Studies done in Zambia using the same data showed that HIV has consistently been associated with mental distress [36].

Educational status has often been identified as a key factor affecting health care utilization. A study in Tanzania revealed that the level of education influenced utilization of health care services [37]. We found education to be a determinant of seeking health care. Educational achievement can be assumed to be associated with an increased awareness of illness, symptoms, and availability of services. Another plausible explanation of this inequity is that the educational level act as a good proxy of socioeconomic position by enhancing the ability to afford the various costs involved. This is also supported by the finding that the wealthy respondents used health services more than the poor, in rural areas. At the time of the survey, most health care service costs were substantially high for the majority of the population. These observations correlate with previous studies in Kenya, Tanzania, and Ghana [38-40].

We found that women utilized health services more often than men in urban areas. This is in agreement with similar studies in the region and internationally [37-39]. There are different explanations of these gender-based differences. One of the explanations is that women are more responsive to symptoms than men because women are more interested in health and have more knowledge in these issues [8].

One of the strengths of this study is that the data were obtained from a survey done among adult men and women in the general population. However, the results from any survey of this type can be expected to be biased to some degree. Non-participation could have introduced bias in the results. In previous publications on HIV prevalence based on the same data, we did not find signs of serious biases since refusal rates were low and those absent might not have substantially differed from the participants in terms of the likelihood of being HIV infected [21,22,27]. For the present analysis, we were primarily concerned about the extent to which non-participation could have biased associations. The magnitude and direction of such biases are difficult to judge. Given that absence is an indication of mobility, we might postulate that non-participants are less likely to use health services. A likely possibility in that case is that the relatively high absence rate among men compared to women might have influenced the estimated gender differences, i.e. the difference might have been underestimated. Reporting bias is another possible source of bias due to the long recall period for reporting visits. Studies often use a recall period of 12 months, although realizing that this will lead to a general underreporting of use (memory bias). Since this is likely to be a general bias across all groups, it is not likely to have significantly biased results from the current study.

The validity of extrapolating the present findings to a larger population is difficult to judge. The population-based survey covered a typical urban population and a rural population that also included very remote populations. The areas surveyed were selected as being typical of the rest of the country in terms of resources and structure of the health care system. Furthermore, the HIV prevalence rates for the selected populations were similar to those observed at the national level [34]. We believe that the major findings of our study can be generalised to a larger population.

## Conclusion

In conclusion, this study revealed substantial socio-economic inequalities in utilization of health facilities in both rural and urban areas. Intervention to improve equality of access to health facilities should be designed in view of the factors found in the study.

## Competing interests

The authors declare that they have no competing interests.

## Authors’ contributions

CZ analysed the data, interpreted the findings and drafted the manuscript. SS assisted in the statistical handling, interpreted the findings, and revised the manuscript. KF designed the survey, participated in the analysis, interpretation of the findings and revision of the manuscript. All authors approved the final version of the manuscript.

## Pre-publication history

The pre-publication history for this paper can be accessed here:

http://www.biomedcentral.com/1472-6963/12/389/prepub

## References

[B1] Gwatkin D Health inequalities and the health of the poor: what do we know? What can we do? Bull World Health Organ 2000 78 1 3 18 10686729 PMC2560590

[B2] Wagstaff A Socioeconomic inequalities in child mortality: comparisons across nine developing countries Bull World Health Organ 2000 78 1 19 29 10686730 PMC2560599

[B3] WHO Making a difference. The world health report 1999 Health for the Millions 1999 25 4 3 5 12295398

[B4] Fryatt B Reaching the poor with health, nutrition and population services: what works, what Doesn't, and Why. Davidson R gwatkin, adam wagstaff and abdo S yazbeck (eds). USA: the world bank Int J Epidemiol 2006 34 4 1109 1110

[B5] Merrick T Focusing on quality and need. The world bank health, nutrition and population sector has been reforming itself to better meet the needs of its clients - the people and governments of the countries it serves Integration 1998 56 26 28 12294062

[B6] Stafford M Nazroo J Popay JM Whitehead M Tackling inequalities in health: evaluating the New deal for communities initiative J Epidemiol Community Health 2008 62 4 298 304 10.1136/jech.2006.058628 18339821

[B7] Narayan D Patel R Schafft K Randemacha A Koch-Schulte S Voices of the poor: can anyone hear us? 2000 New York: Oxford university press

[B8] Pritchet L Summers L Wealthier is healthier J Hum Resour 1996 31 841 868 10.2307/146149

[B9] Ruger J Ethics and governance of global health inequalities J Epidemiol Community Health 2006 60 998 1003 10.1136/jech.2005.041947 17053290 PMC2465483

[B10] Whitehead M The concepts and principles of equity and health Int J Health Serv 1992 22 3 429 445 10.2190/986L-LHQ6-2VTE-YRRN 1644507

[B11] Grand L Equity, health and health care. Social justice research 1987 Soc Justice Res 1987 1 3 257 274 10.1007/BF01047663

[B12] Smaje C Ethnicity, equity and the use of health services in the British NHS Soc Sci Med 1997 45 3 485 496 10.1016/S0277-9536(96)00380-2 9232742

[B13] Alleyne GA Casas JA Castillo-Salgado C Equality, equity: why bother? Bull World Health Organ 2000 78 1 76 77 10686736 PMC2560607

[B14] Wagstaff A Economics, health and development: some ethical dilemmas facing the world bank and the international community J Med Ethics 2001 27 4 262 267 10.1136/jme.27.4.262 11479358 PMC1733428

[B15] Cummings KM Becker MH Maile MC Bringing the models together: an empirical approach to combining variables used to explain health actions J Behav Med 1980 3 2 123 145 10.1007/BF00844986 7420418

[B16] Andersen R National health surveys and the behavioral model of health services use Medical Care 2008 46 7 647 653 10.1097/MLR.0b013e31817a835d 18580382

[B17] MOH National strategic health plan 2006–2010 2005 Lusaka: Ministry of Health

[B18] CBoH Health institution in zambia: a listing of health facilities according to levels and locations 2002 Lusaka: Central Board of Health

[B19] Hjortsberg C Why do the sick not utilise health care? The case of Zambia Health Econ 2003 12 9 755 770 10.1002/hec.839 12950094

[B20] Makinen M Inequalities in health care use and expenditure: empirical data from eight developing countries and countries in transition Bull World Health Organ 2000 78 1 55 65 10686733 PMC2560608

[B21] Michelo C Sandoy IF Dzekedzeke K Siziya S Fylkesnes K Steep HIV prevalence declines among young people in selected Zambian communities: population-based observations (1995–2003) BMC Publ Health 2006 6 279 10.1186/1471-2458-6-279 PMC1660545 17096833

[B22] Fylkesnes K Ndhlovu Z Kasumba K Mubanga Musonda R Sichone M Studying dynamics of the HIV epidemic: population-based data compared with sentinel surveillance in Zambia AIDS 1998 12 10 1227 1234 10.1097/00002030-199810000-00015 9677172

[B23] SPSS SPSS statistics for windows,version 12.0 2003 Chicago: SPSS Inc

[B24] StataCorp Stata statistical software: release 9 2005 College Station, TX: StataCorp LP

[B25] Wannamethee G Shaper A Self-assessment of health status and mortality in middle-aged British men Int J Epidemiol 1991 20 1 239 245 10.1093/ije/20.1.239 2066228

[B26] Thomas C Kelman HR Kennedy GJ Ahn C Yang CY Depressive symptoms and mortality in elderly persons J Gerontol 1992 47 2 S80 S87 10.1093/geronj/47.2.S80 1538079

[B27] Siziya S Fylkesnes K Impact of HIV infection on self-rated health in a high-prevalence population with low awareness of own HIV statu Norsk Epidemiologi 2005 15 165 173

[B28] Mossey J Shapiro E Self-rated health: a predictor of mortality among the elderly Am J Public Health 1982 72 8 800 808 10.2105/AJPH.72.8.800 7091475 PMC1650365

[B29] Fylkesnes K Determinants of health care utilization-visit and referrals Scand J SocMed 1993 21 1 40 50 10.1177/140349489302100107 8469943

[B30] Fylkesnes K Johnsen R Olav F The tromsø study: factors affecting patient-initiated and provider- initiated use of health care services Social Health Illness 1992 14 2 275 292 10.1111/1467-9566.ep11343713

[B31] Filmer D Pritchett L Estimating wealth effects without expenditure data–or tears: an application to educational enrollments in states of India Demography 2001 38 1 115 132 11227840 10.1353/dem.2001.0003

[B32] WHO Public spending on health care in africa: do the poor benefit? B WORLD HEALTH ORGAN 2000 78 1 66 74 PMC2560601 10686734

[B33] Brown M Using gini-style indices to evaluate the spartial patterns of health practitioners: theoretical considerations and application based on alberta Soc Sci Med 1994 38 9 1243 1256 10.1016/0277-9536(94)90189-9 8016689

[B34] Michelo C Sandoy I Fylkesnes K Marked HIV prevalence declines in higher educated young people: evidence from population-based surveys (1995–2003) in Zambia AIDS 2006 20 7 1031 1038 10.1097/01.aids.0000222076.91114.95 16603856

[B35] Fylkesnes K Siziya S A randomized trial on acceptability of voluntary HIV counselling and testing Trop Med Int Health 2004 9 5 566 572 10.1111/j.1365-3156.2004.01231.x 15117300

[B36] Chipimo P Fylkesnes K Mental distress in populations with high HIV prevalence: impact of HIV and social factors BMC Publ Health 2009 9 298 10.1186/1471-2458-9-298 PMC2744699 19689799

[B37] Wyss K Kilima P Utilization of Government and private health services in Dar es Salaam East Afr Med J 1996 73 6 357 363 8840594

[B38] Nyamonga I Health care switching behavior of malaria patients in Kenya rural community Soc Sci Med 2002 54 3 377 386 10.1016/S0277-9536(01)00036-3 11824914

[B39] Masatu M Kvåle G Klepp K Health services utilizations among secondary school students in Arusha region, Tanzania East Afr Med J 2001 78 6 300 307 12002108 10.4314/eamj.v78i6.9023

[B40] Asenso-Okyere WK Anum A Osei-Akoto I Adukonu A Cost recovery in Ghana: are there any changes in health care seeking behaviour? Health Policy Plan 1998 13 2 181 188 10.1093/heapol/13.2.181 10180407

